# Visceral adipose tissue is an independent predictor and mediator of the progression of coronary calcification: a prospective sub-analysis of the GEA study

**DOI:** 10.1186/s12933-023-01807-6

**Published:** 2023-04-03

**Authors:** Neftali Eduardo Antonio-Villa, Juan Gabriel Juárez-Rojas, Rosalinda Posadas-Sánchez, Juan Reyes-Barrera, Aida Medina-Urrutia

**Affiliations:** Departamento de Endocrinología, Instituto Nacional de Cardiología Ignacio Chávez, Juan Badiano 1, Col. Sección XVI, C.P. 14080 Ciudad de Mexico, Tlalpan México

**Keywords:** Cardiovascular risk, Coronary artery calcium progression, Visceral abdominal fat, Dysfunctional adiposity

## Abstract

**Background:**

Coronary artery calcium (CAC) improves cardiovascular event prediction. Visceral adipose tissue (VAT) is a cardiometabolic risk factor that may directly or through its related comorbidities determine the obesity-related risk. A clinical VAT estimator could allow an efficient evaluation of obesity-related risk. We aimed to analyze the effect of VAT and its related cardiometabolic risk factors on CAC progression.

**Methods:**

CAC was quantified at baseline and after 5 years by computed tomography (CT), determining its progression. VAT and pericardial fat were measured by CT and estimated by a clinical surrogate (METS-VF). Considered cardiometabolic risk factors were: peripheral insulin resistance (IR), HOMA-IR, adipose tissue IR (ADIPO-IR), and adiponectin. Factors independently associated to CAC progression were analyzed by adjusted Cox proportional hazard models, including statin use and ASCVD risk score as covariates. We performed interaction and mediation models to propose possible pathways for CAC progression.

**Results:**

The study included 862 adults (53 ± 9 years, 53% women), incidence CAC progression rate: 30.2 (95% CI 25.3–35.8)/1000 person-years. VAT (HR: 1.004, 95% CI 1.001–1.007, p < 0.01) and METS-VF (HR: 1.001, 95% CI 1.0–1.001, p < 0.05) independently predicted CAC progression. VAT-associated CAC progression risk was evident among low-risk ASCVD subjects, and attenuated among medium–high-risk subjects, suggesting that traditional risk factors overcome adiposity in the latter. VAT mediates 51.8% (95% CI 44.5–58.8%) of the effect attributable to IR together with adipose tissue dysfunction on CAC progression.

**Conclusions:**

This study supports the hypothesis that VAT is a mediator of the risk conferred by subcutaneous adipose tissue dysfunction. METS-VF is an efficient clinical surrogate that could facilitate the identification of at-risk adiposity subjects in daily clinical practice.

**Supplementary Information:**

The online version contains supplementary material available at 10.1186/s12933-023-01807-6.

## Introduction

Coronary artery disease (CAD) is one of the leading causes of morbidity and mortality worldwide [1]. Although CAD could result in cardiovascular events, it is often asymptomatic and undiagnosed within clinical practice. Non-contrasted computed tomography (CT) is a non-invasive and sensitive method that quantifies coronary artery calcium (CAC) and allows the identification of subclinical atherosclerosis [2]. Moreover, CAC assessed by the Agatston score improves the prediction of cardiovascular events in asymptomatic individuals, particularly among those classified as intermediate risk by traditional cardiovascular risk estimators [3]. Therefore, an increase in the Agatston score is a good predictor of atherosclerosis progression and CAD-related mortality [4].

Although large-scale epidemiological studies have shown that body mass index (BMI) correlates with an increased burden of cardiovascular risk factors (CRF) such as hypertension, dyslipidemia, and diabetes, [5, 6] it is not consistent for all subjects, [7, 8] suggesting that BMI may not be a good indicator of adiposity-related cardiovascular risk [9]. However, post-mortem studies [10] and other analyses carried out in prospective cohorts in which the development of imaging techniques has allowed the identification and quantification of ectopic fat deposits suggest that visceral abdominal fat (VAT) [11, 12] and pericardial fat [13, 14] are responsible for cardiovascular risk related to adiposity. On the other hand, it has also been suggested that subcutaneous adipose tissue dysfunction, characterized by insulin resistance, low plasma adiponectin, and increased ectopic fat deposits, is the initial damage that promotes CAD development [15, 16]. Moreover, it has been observed that body fat is a pathophysiological modulator related to the incidence and progression of CAC [17]. Direct measurement of ectopic fat requires expensive equipment, inaccessible to clinical practice. Therefore, some clinical estimators of VAT that combine anthropometric and biochemical parameters have been proposed [18]. METS-VF is a clinical VAT estimator developed and validated in Mexican population [19].

Considering that the causal mechanisms underlying CAD progression related to adipose-tissue are still under study, and that VAT estimators would allow the early and efficient identification of individuals with increased cardiovascular risk related to body fat within clinical practice. This study aimed: (1) to evaluate the effect of VAT and adiposity function markers on the risk of CAC progression; (2) asses the interactive and causal-mediation effect of VAT with markers of adipose tissue dysfunction and their possible role in CAC progression; and (3) explore whether the estimation of VAT, using the clinical surrogate METS-VF, enables the recognition of the risk conferred by VAT on CAC progression.

## Methods

### Study design

**Fig. 1:**
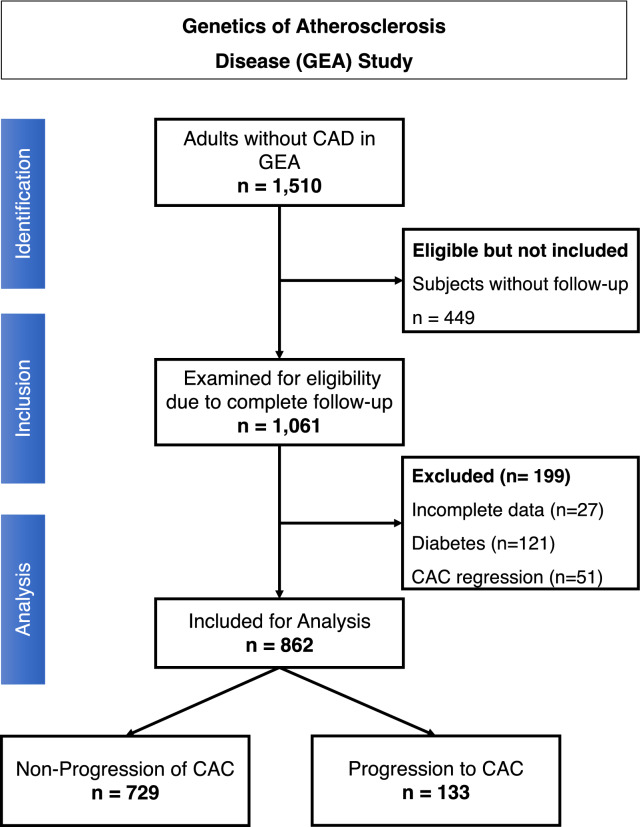
STROBE flow-chart of our studied population from the Genetics of Atherosclerosis Disease (GEA) study. *CAD* coronary artery disease, *CAC* coronary artery calcium

The present study is a sub-analysis of the Genetics of Arteriosclerotic Disease (Spanish acronym GEA) study carried out at the Instituto Nacional de Cardiología Ignacio Chávez, in Mexico City. The complete methodology and preliminary results are reported elsewhere [20]. The GEA study is a prospective cohort of a Mexican mestizo population designed to examine the genomic bases of premature CAD and its association with traditional and emerging cardiovascular risk factors. The baseline recruitment was conducted between 2008 and 2012 on a convenience sample of residents of Mexico City and the metropolitan area. The initial cohort included 1510 non related adults (≥ 20 years) with no personal or family history of premature CAD. Five years later, the participants were invited via telephone calls, telegrams, and personal contacts to participate in the follow-up phase of the study, conducted between 2013 and 2019. The response rate was 70.3% (n = 1061). For the present analysis, we excluded participants with incomplete CT data (n = 27), CAC regression (n = 51), or initial diagnosis of diabetes (n = 121) for a total of 862 participants (Fig. 1). The institutional ethics committee approved the baseline study (Protocol No. 09–646) and the follow-up analysis (Protocol No. 22–1297). The project followed the guidelines of the 1975 Helsinki declaration, and each participant gave signed informed consent. The writing of this work adheres to the STROBE reporting guidelines for cohort studies (Additional file).

### Primary outcome definition

The primary outcome of the present analysis was CAC progression. Although there is no consensus regarding the CAC progression definition, the present study used the Hokanson et al*.* definition since it considers inter-scanner variability in a wide range of CAC values. Hokanson et al*.* define CAC progression as a difference ≥ 2.5 cm^2^ of the square root of CAC at follow-up minus the square root of CAC at baseline [21].

### Analyzed variables

#### Clinical evaluation

At the baseline and follow-up visits, standardized questionnaires were applied by previously trained personnel in order to investigate demographic variables, family medical history, alcohol intake, tobacco, and drug use [20]. Anthropometric measurements recorded by a certified nutritionist included weight, height, and waist circumference in centimeters. BMI was defined as weight in kilograms, divided by height in square meters. Blood pressure was measured after 10 min in a seated position.

#### Biochemical parameters

Fasting venous blood samples were obtained. Plasma concentrations of glucose, total cholesterol (TC), triglycerides (TG), and high-density lipoprotein cholesterol (HDL-C) were determined by enzymatic-colorimetric reagents (Roche/Hitachi, Germany). Serum free fatty acid (FFA) concentration was measured with Wako Diagnostics Kit (Chuo-Ku Osaka Japan), on a Hitachi 902 autoanalyzer (Hitachi LTD, Tokyo Japan). Coefficients of variation were less than 3% for all variables. Low-density lipoprotein cholesterol (LDL-C) was estimated by Friedewald formula [22]. Serum adiponectin was evaluated by ELISA (R and D systems, Minneapolis USA, Quantine Kit), coefficient of variation < 10%. Total high sensitive C-reactive protein (hs-CRP) was determined by immunonephelometry on a BN ProSpec nephelometer (Dade Behring, Marburg Hesse, Germany), coefficient of variation < 6%. Serum insulin was quantified by radioimmunoassay (Millipore RIA ST Charles, Missouri, US), coefficient of variation < 6.8%.

#### Tomographic measurements

VAT, subcutaneous adipose tissue (SAT), pericardial fat volume (PFV) and CAC were quantified by CT scan. For the present analyses, we used VAT, SAT and PFV baseline measurements. To establish CAC progression baseline and follow-up CAC scores were analyzed. CT images were quantified in a 64-channel helical multidetector tomograph (Somaton Sensation, Siemens, Malvern, PA, USA) until 2009, or in a 256-channel tomograph (Somaton Sensation, Siemens, Malvern, PA, USA) after 2009. A specialized radiologist interpreted the scans at a Leonardo workstation (Siemens, Forchheim, Germany) equipped with a CAC score analysis program (CaScoring, Siemens, Forchheim, Germany). VAT was quantified as described by Kvist et al. [23]. PFV was measured in a section that included 15 mm above and 30 mm below the origin of the left main coronary artery. This heart region includes the fat located around the proximal coronary arteries (left main coronary, left anterior descending, right coronary, and circumflex arteries), pericardial fat was manually outlined in every consecutive slice, and its volume was obtained using a threshold attenuation between − 190 and − 30 Hounsfield Units (HU) [14]. The CAC was evaluated according to Agaston’s method [24]. The reanalysis of 20 chest and abdomen images was used to obtain the inter-observer variability, finding an intra-class correlation of 0.99 for all measurements.

### Cardio-metabolic risk factors definitions

Overweight was defined as a BMI between 25 and 29.9 kg/m^2^ and obesity as a BMI ≥ 30 kg/m^2^. Consistent with the International Diabetes Federation (IDF) criteria for Mexican population, central obesity was defined as a waist circumference ≥ 90 cm in men and ≥ 80 cm in women [25]. We considered smoking when the participant reported having smoked ≥ 1 cigarette/day in the previous year. Hypertension was defined when the participant recorded values ≥ 140 mmHg for systolic blood pressure or ≥ 90 mmHg for diastolic blood pressure or used antihypertensive drugs [26]. Abnormal fasting glucose was defined as fasting glycaemia values between 100 and 125 mg/dL. Dyslipidemia was defined as a composite of having hypercholesterolemia, hypertriglyceridemia, and low HDL-C values according to LDL-C ≥ 130 mg/dL, TG ≥ 150 mg/dL, and HDL-C ≤ 40 mg/dL for men or ≤ 50 mg/dL for women, respectively. We used the ASCVD (atherosclerotic cardiovascular disease) score, defined by the American Heart Association (AHA) and the American College of Cardiology (ACC), estimated with baseline variables, to enclose traditional cardiovascular risk [27]. Subjects with an ASCVD < 5 were classified as low risk, those between 5 and 7.5 as intermediate risk, and an ASCVD ≥ 7.5 as high risk.

***Systemic insulin resistance: ***was estimated by the homeostasis model for IR formula: ${\text{HOMA}} - {\text{IR}}\, = \,\left( {{\text{fasting insulin }}\left[ {\mu {\text{U}}/{\text{mL}}} \right] \, *{\text{ fasting glucose }}\left[ {{\text{mmol}}/{\text{mL}}} \right]} \right) \, /{ 22}.{5})$ [28].

***Adipose tissue insulin resistance: ***was calculated as ${\text{Adipo}} - {\text{IR}}\, = \,{\text{FFA }}\left[ {{\text{mmol}}/{\text{L}}} \right] \, *{\text{ Insulin }}\left[ {\mu {\text{U}}/{\text{L}}} \right])$ [29].

#### VAT clinical estimation

We included the metabolic score for visceral fat estimation (METS-VF) as clinical estimator of VAT.

$$ {\text{METS}} - {\text{VF}} = { 4}.{466 } + \, 0.0{11}\left[ {\left( {{\text{LnMETS }} - {\text{IR}}} \right)^{{3}} } \right] \, + { 3}.{239}\left[ {{\text{Ln}}\left( {{\text{waist circumference}}/{\text{height}}} \right)^{{3}} } \right] \, + \, 0.{319}\left( {\text{male sex}} \right) \, + \, 0.{594}\left[ {{\text{Ln}}\left( {{\text{age}}} \right)} \right]) $$

The exponential transformation of METS-VF reflects a estimation of VAT in grams [19].

#### Thresholds of VAT and IR

Increased VAT and insulin resistance (IR) were defined with the 75th percentile value (p ≥ 75) using a sample of the same GEA study, including subjects without cardiovascular risk factors. The thresholds previously identified were: p75 > VAT = 127 cm^2^ in women and 152.7 cm^2^ in men. For p75 > HOMA-IR: > 3.58 in women and > 3.12 in men [30].

### Statistical analyses

According to the distribution of the continuous data, we displayed the variables as means (standard deviation) or medians (interquartile range) determined by the Anderson–Darling normality test, and categorical variables as frequency and absolute proportions. The incidence rate of CAC progression was estimated by the Wilson approach method, with a 95% confidence interval (CI), using the *epiR* package [31]. Baseline study cohort characteristics stratified by the incident and non-incident CAC progression were made with a Student-t test, Wilcoxon-Rank test, or Chi-Squared test, as appropriate. All statistical analyses were performed in *R Studio* (Version 4.1.2). A value of p < 0.05 was the statistically significant threshold.

#### Correlates of METS-VF with VAT measurements

First, we evaluate the correlation of METS-VF with anthropometric and tomographic VAT measurement, using Spearman correlation coefficients and the exponential transformation of METS-VF. A correlogram matrix was fitted using the *ggplot2* R package [32]. Then, to evaluate whether METS-VF identifies subjects classified with excessive VAT (≥ p75), we performed an AUROC analysis.

#### Prediction of CAC progression attributable to adiposity and VAT

To evaluate the role of adiposity and VAT measurements as predictors of CAC progression, Cox proportional hazard regression models were fitted using the Breslow method. The first model (Model 1) was adjusted by statin usage and ASCVD score, and the second model (Model 2) for covariates in Model 1 plus HOMA-IR, adiponectin, C-reactive protein, hypertension status and dyslipidemia status. A simulation-based plot was fitted for the second model to visualize the dose-relationship association of adiposity and VAT measurements with CAC progression using the *simpH* R package [33]. The goodness of fit was tested using the likelihood ratio, C-statistic, and the Bayesian Information Criteria (BIC). Multicollinearity was tested using the variance inflation factor (VIF) for each fitted model with two or more variables. The proportional hazard assumption was tested using the Schoenfeld residuals. All the models and diagnostic statistics were fitted using the *survival* R package and are presented in the Additional file 1: Table S4 [34].

#### Sensitivity analysis

We hypothesize that ectopic deposits of visceral fat are driven by similar pathophysiological mechanisms across different compartments. Therefore, to support the hypothesis that VAT acts as a marker of CAC progression, we performed a secondary analysis using PFV within a subsample of the GEA study as another metric of ectopic visceral fat. We used Cox proportional hazard regression models adjusted for the same covariates mentioned for model 1 and model 2.

#### Modifiers of the effect of VAT on CAC progression

We hypothesize that the relationship between VAT and CAC progression could be modified by baseline cardiovascular risk, systemic or adipose tissue IR, or adiponectin. Hence, we perform interaction models for VAT and METS-VF considering ASCVD score (as a proxy for cardiovascular risk), HOMA-IR (as a proxy for systemic IR), ADIPO-IR (as a proxy for adipose tissue IR), and adiponectin (as a proxy for adipose tissue dysfunction) as interaction terms. Models and interaction coefficients are presented in the Additional file 1: Tables S2, S3.

#### Causal mediation analysis

Previous studies have reported the mediation capacity of VAT on vascular health and the risk of arterial hypertension [35]. Here, we explore whether VAT may act as a mediator of the risk conferred by systemic and adipose tissue IR along with adiponectin on CAC progression. To propose a causal-mediation pathway, three-step-way Cox regression models were fitted to support a possible mechanism. Step 1 included the univariate relation between HOMA-IR, ADIPO-IR, and adiponectin against the risk of CAC progression. Step 2 included a double combination of the previously mentioned factors (HOMA-IR plus Adiponectin, HOMA-IR plus ADIPO-IR, Adiponectin plus ADIPO-IR). Then, step 3 included the double combination of the covariates included in Step 2 adjusted for VAT. Finally, we integrated a diagram that could link the pathophysiological mechanism and performed causally ordered model-based mediation analyses using beta coefficients extracted from Cox proportional risk regression models to test the mediation mechanism. To assess and obtain a 95% confidence interval, we used a bias-corrected accelerated non-parametric bootstrap over 1,000 resamples. All mediation analyses were performed using the *mediation R* package [36].

## Results

### Baseline characteristics

The baseline characteristics of the studied subjects, stratified by CAC progression status, are shown in Table 1. Briefly, the studied sample consisted of 47% men, with a mean age of 52 ± 9 years and a follow-up time of 4.8 (range, 4.5–5.3) years. In the overall sample, we observed a high prevalence of overweight (49%), obesity (29.2%), central obesity (80.3%), and increased VAT (55.5%). At baseline, 176 subjects (20.4%) had coronary calcification, of whom 116 (66%) had CAC > 10 HU, 43 (24.4%) CAC > 100 HU, and only 10 (5.7%) had CAC > 400 HU.

**Table 1:** Baseline study cohort characteristics by CAC progression

	Total N = 862	No progression N = 729	Progression N = 133	*p*^c^
Clinical variables				
Age (years)	52.9 ± 8.9	51.9 ± 8.7	58.3 ± 8.2	< 0.0001
Male (%)	47.2	42.5	72.9	< 0.0001
Follow-up (years)	4.8 (4.5–5.3)	4.7 (4.5–5.2)	4.8 (4.6–5.7)	< 0.05
Smoking (%)	17.3	17.6	15.6	NS
Systolic BP (mmHg)	114.7 ± 16.0	113 ± 15.0	124.7 ± 18.0	< 0.0001
Diastolic BP (mmHg)	71.2 ± 9.1	70.0 ± 8.6	75.8 ± 10.3	< 0.0001
Antihypertensive use (%)	17.6	17.6	18.0	NS
Statin use (%)	10.0	9.1	15.0	< 0.05
Biochemical variables				
TC (mg/dL)	194.0 ± 37.1	193.5 ± 36.4	197.2 ± 40.9	NS
LDL-C (mg/dL)	119.1 ± 32.1	118.7 ± 31.7	121.2 ± 33.9	NS
TG (mg/dL)	146 (107–201)	142 (106–197)	157 (118–212)	0.07
HDL-C (mg/dL)	46.5 ± 13.4	46.8 ± 13.4	44.8 ± 13.2	NS
Glucose (mg/dL)	90.1 ± 9.2	89.6 ± 9.1	93.4 ± 9.4	< 0.0001
Insulin resistance variables				
HOMA-IR	3.6 (2.6–5.2)	3.5 (2.5–5.0)	4.6 (2.9–5.8)	< 0.0001
METS-IR	43.7 ± 9.2	43.4 ± 9.2	45.4 ± 9.1	< 0.05
ADIPO-IR	9.2 (6.0–13.9)	8.9 (5.9–13.5)	10.4 (6.4–15.9)	NS
Adiponectin (μg/mL)	8.1 (5.0–12.9)	8.4 (5.1–13.4)	6.7 (4.2–11.3)	< 0.01
C-reactive protein (mg/L)	1.5 (0.8–3.0)	1.5 (0.8–3.0)	1.6 (1.0–2.8)	NS
Adiposity measures				
BMI (Kg/m^2^)	28.2 ± 4.3	28.1 ± 4.3	28.9 ± 4.1	0.06
WC (cm)	94.0 ± 11.5	93.2 ± 11.5	97.8 ± 10.6	< 0.0001
VAT (cm^2^)	145 (106–185)	137 (103–176)	175 (136–212)	< 0.0001
SAT (cm^2^)	283 (208–354)	286(210–362)	262 (199–338)	0.08
PVF (cm^3^)^b^	41.8 (29.2–56.5)	40.7 (28.2–56.0)	46.1 (32.9–63.9)	0.01
METS-VF	7.0 ± 0.5	6.9 ± 0.5	7.2 ± 0.4	< 0.0001
Cardiovascular risk				
ASCVD score	3.1 (1.3–6.9)	2.5 (1.2–5.7)	7.3 (4.6–12.9)	< 0.0001
Baseline Agatston score^a^	0 (0–935)	0 (0–673)	11 (0–935)	< 0.0001
Follow-up Agatston score^a^	0 (0–1792)	0 (0–768)	75 (6–1792)	< 0.0001

Data are expressed as mean ± standard deviation, median (interquartile range), percentage or ^a^median (min–max)

*BMI* body mass index, *VAT* visceral adipose tissue, *SAT* subcutaneous adipose tissue, ^b^*PVF* perivascular fat (n = 628) *METS-VF* metabolic score for visceral fat estimation, *TC* total cholesterol, *LDL-C* low density lipoprotein cholesterol, *TG* triglycerides, *HDL-C* high density lipoprotein cholesterol, *HOMA-IR* homeostatic model for insulin resistance, *METS-IR* metabolic score for insulin resistance estimation, *ADIPO-IR* adipose tissue insulin resistance, *BP* blood pressure, *ASCVD* atherosclerotic cardiovascular disease risk score

^c^t-student test, Mann Whitney, or Chi-square, as correspond

### Characterization of subjects with CAC progression

**Fig. 2:**
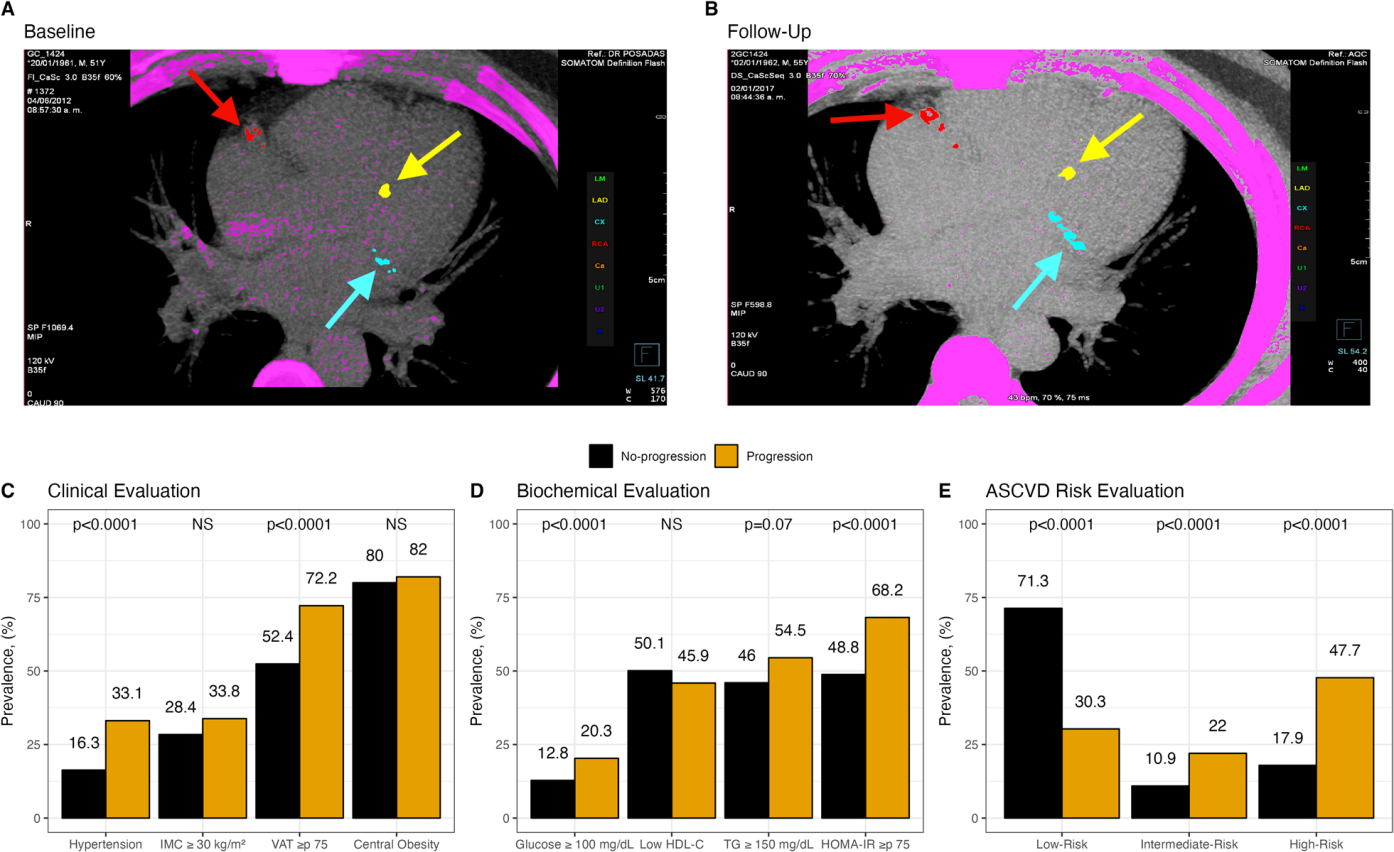
Tomographic evaluation of a 51-year-old male participant, BMI = 31.5 kg/m^2^ and 181 cm^2^ VAT at baseline (panel A) and follow-up (panel B) showing CAC progression. Arrows point to the right coronary artery (RCA), left anterior descending (LAD), and circumflex (CX) artery. Panels C, D, and E show the prevalence of clinical and biochemical variables and ASCVD risk classification, stratified by CAC progression. Differences were analyzed using the chi-square test

We observed CAC progression in 133 participants (15.4%), incidence of 30.2 (95% CI 25.3–35.8) per 1000 person-years-follow-up. Male sex, age, and statin use were higher among progressors (Table 1). A schematic case displaying the tomographic progression of CAC is presented in Fig. 2, panel A (baseline CAC measurement), panel B (follow-up CAC measurement). Although BMI was similar between subjects with or without progression, waist circumference, VAT, PVF, and METS-VF were higher among progressors (Table 1, Fig. 2 panel C). CAC progressors had higher prevalence’s of hypertension (16.3% vs. 33.1%, p < 0.0001), abnormal fasting glucose (12.8% vs. 20.3%, p < 0.0001), and systemic IR (48.8% vs. 68.2%, p < 0.0001), Fig. 2, panel C and D. Regarding baseline cardiovascular risk, assessed by ASCVD score (Fig. 2, panel E) 12.6% were classified as intermediate risk (no progression: 10.9% vs. progression: 22.0%, p < 0.0001) and 22.4% as high risk (no progression: 17.9% vs. progression: 47.7%, p < 0.0001).

### Correlates of CT-VAT with METS-VF

The exponential value of METS-VF, which estimates VAT in grams, was the variable that best correlated with the direct CT-VAT quantification (r = 0.752, 95% CI 0.72–0.78, p < 0.0001). In addition, METS-VF identified subjects with high VAT (≥ p75), with and optimal AUC (0.84; 95% CI 0.81–0.87, p < 0.0001). (Additional file 2: Fig S1).

### Impact of adiposity measures and VAT on CAC progression

**Fig. 3:**
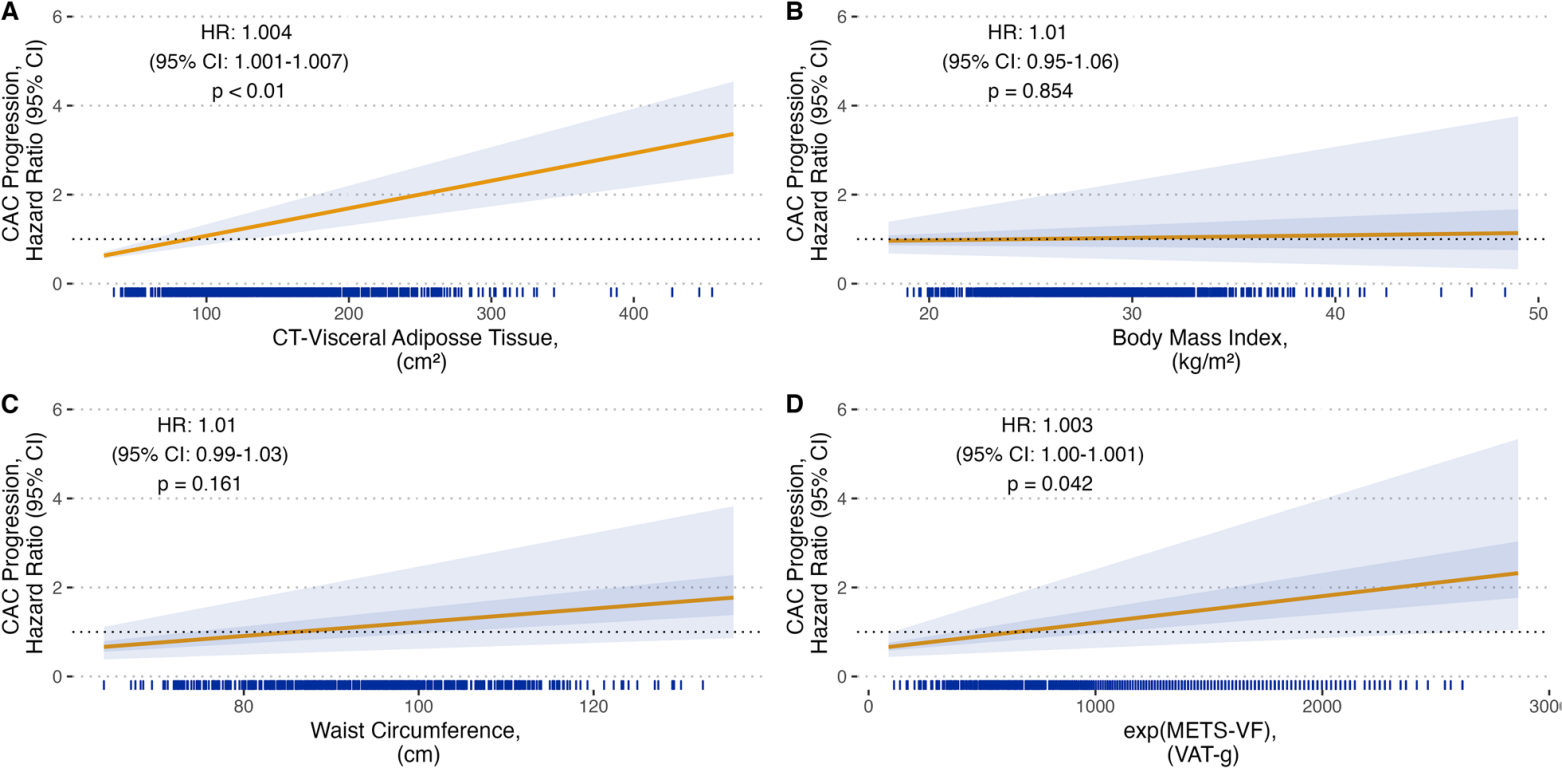
Simulation-based plot of Hazard ratio to assess the dose-relationship of adipose tissue markers and CAC progression. The plot was made using the simpH R package

Cox proportional hazard models were used to analyze the effect of adiposity measures on the risk of CAC progression. In univariate models (Additional file 1: Table S1), BMI was not associated with the risk of progression. Conversely, central adiposity metrics were predictor variables (VAT: HR = 1.007 [95% CI 1.004–1.009], p < 0.0001; waist circumference: HR = 1.03, [95% CI 1.02–1.04], p < 0.0001). The association with waist circumference persisted after adjusting for ASCVD and statin use (model 1) but decreased after adjusting for HOMA-IR, adiponectin, C-reactive protein, hypertension status and dyslipidemia status (model 2). Only VAT and METS-VF were predictors of CAC in the fully adjusted models (Fig. 3, Additional file 1: Table S1). To support the hypothesis that VAT predicts CAC progression, we performed a sensitivity analysis using PFV available within 628 individuals. The unadjusted model showed that PFV predicts CAC progression (HR = 1.012, [95% CI 1.006–1.019], p < 0.01). The association persisted in Model 1 (HR = 1.010, [95% CI 1.003–1.017], p = 0.007). However after adjusting for covariates in Model 2 the association was attenuated (HR = 1.008, [95% CI 0.998–1.015], p = 0.095).

### Impact of markers of adipose tissue dysfunction upon the effect of VAT on CAC progression

We constructed interaction and mediation models to determine if ASCVD, HOMA-IR, ADIPO-IR, and adiponectin, modifies the effect of VAT on CAC progression.

#### Interaction analysis

This analysis shows that the effect of VAT on CAC progression is dependent on the ASCVD score. The risk of CAC progression attributable to VAT is higher among subjects classified with a low ASCVD score (HR = 1.008, 95% CI 1.004–1.012, p < 0.0001). However, the VAT effect was attenuated among subjects with intermediate (HR = 1.003, 95% CI 0.99–1.008, p = 0.27) to high-risk ASCVD score (HR = 1.003, 95% CI 0.99–1.006, p = 0.17; Additional file 1: Table S2). These results suggest that traditional cardiovascular risk factors surpassed the effect of VAT on CAC progression. Conversely, IR and adiponectin did not interact with the risk of VAT on CAC progression. These analyzes showed similar results when the METS-VF values were used (Additional file 1: Table S3).

#### Causal mediation analysis

**Fig. 4:**
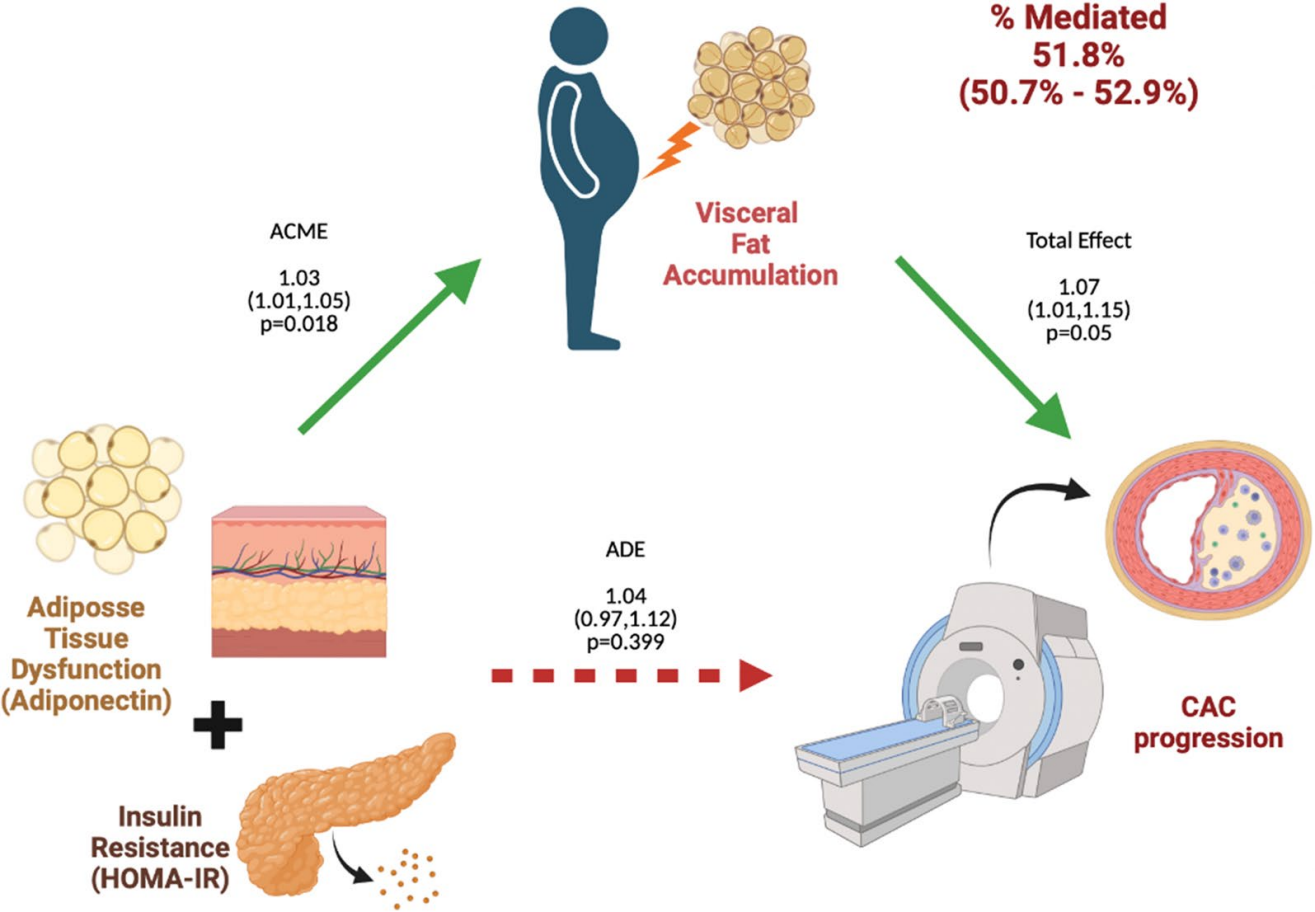
Model-based causal mediation analyses to evaluate mediation capacity of VAT on the risk of adipose tissue dysfunction and insulin resistance for CAC progression. *ADE* attributable direct effect, *ACME* attributable causal mediation effect. Mediation models were fitted using the beta coefficients extracted from Cox proportional Hazard regression models. Confidence intervals were obtained using bias-corrected accelerated non-parametric bootstrap over 1,000 resamples

As a next objective, we investigated whether VAT could mediate CAC progression through systemic IR (HOMA-IR), adipose tissue IR (ADIPO-IR) and adipose tissue dysfunction (adiponectin). In a first step, we evaluate the univariate relation between HOMA-IR, ADIPO-IR, and adiponectin with CAC progression. We observed that HOMA-IR and adiponectin predict CAC progression, while ADIPO-IR had a marginal effect (Additional file 1: Table S4, Step 1). In a second step, the double-combination of the previously mentioned factors was fitted, observing that adiponectin was the strongest predictor. Finally, as a third step, the double-combination in steep 2 was VAT adjusted, founding that adiponectin had a marginal effect, while VAT was the strongest predictor, supporting the hypothesis that VAT could mediate the effect of adipose dysfunction markers explored (Additional file 1: Table S4). We then adjusted these models for covariates included in model 2 and observed that the effect sustained (Additional file 1: Table S5). Based on the previous approach, a diagram displaying the potential pathophysiological mechanisms of VAT mediation on CAC progression was constructed (Fig. 4). This diagram proposes that there is a total effect of IR and adiponectin (adipose tissue dysfunction markers) on CAC progression (β_TE_ = 1.07, 95% CI 1.01–1.15, p = 0.05), which is indirectly driven by VAT accumulation (β_ACME_ = 1.03, 95% CI 1.01–1.05, p = 0.02). This model suggests that VAT mediates over 51.8% (95% CI 44.5–58.8%) of the effect attributable to IR in conjunction with adiponectin on CAC progression.

## Discussion

In a prospective analysis of the GEA study, we identified that VAT is an independent predictor for CAC progression, with this risk being further confirmed by estimates of PFV. Moreover, by an ASCVD-VAT interaction analysis, we found that participants classified as low-ASCVD risk had an evident increased risk of CAC progression, which was attenuated among medium to high-risk subjects. Furthermore, mediation models showed that VAT mediates 51.8% of the risk of CAC progression conferred by systemic IR and adiponectin. Finally, the clinical surrogate of VAT, METS-VF, was analyzed, founding that this is a good estimator of the cardiovascular risk associated with VAT.

### Overview of the cardiovascular implications of VAT

The CAC score is an advantageous, non-invasive method that identifies asymptomatic subjects at risk of cardiovascular morbi-mortality [2, 3]. CAC impact on CAD incidence and mortality is twice as much for Hispanics than for Caucasians, [37] suggesting ethnicity inherent factors could have a modifying effect. Thus, the study of CAC progression in the Mexican-Mestizo cohort may be critical since it is characterized by a high prevalence of body mass excess, IR, low adiponectin, and ectopic fat depots [30, 38]. Several studies have shown that the CAC score improves the risk estimated by cardiovascular risk algorithms such as ASCVD [39, 40]. However, these algorithms do not consider adiposity, probably due to the inconsistent association of general adiposity measures as cardiovascular risk factors [9]. The interaction models performed in the present study showed that VAT-associated risk was evident among low-risk ASCVD score subjects, in whom VAT could be used to identify incipient cardiovascular risk and to reconsider early strategies in preventive care medicine; however, the impact of VAT on CAC progression was attenuated among medium to high-risk subjects, suggesting that traditional cardiovascular risk factors may be more critical than adiposity among them.

### Physiopathological mechanism related to the association of VAT with CAC progression

Epidemiological studies, [11, 41] and prospective cohorts [18] have shown that VAT [42–44] and PVF [45] are precursors of CAC progression and cardiovascular events, even in asymptomatic subjects. These studies support the present analysis showing VAT and PVF as associates of CAC progression. The physiopathological pathway which explains our findings are linked with several hypothesis. Previous studies propose the loss of capacity of SAT to store fat as the first step in adipose tissue damage, which favors ectopic fat deposition [9, 15, 35, 45]. This is also accompanied by low adiponectin serum concentrations [30, 46]. In line with these findings, in the present study, progressor subjects had lower values of circulating adiponectin than non-progressors. Therefore, adiponectin could be the link between adipose tissue dysfunction, IR, and atherosclerosis progression [47]. In addition, multiple studies have shown that VAT excess is associated with a higher release of fatty acids into the portal circulation, promoting non-alcoholic fatty liver disease development as well as hepatic and peripheral IR [12, 48, 49]. The present study found a marginal increase in the ADIPO-IR index and a significant increase in the HOMA-IR in subjects with CAC progression. Furthermore, by performing a causal mediation analysis, we found that VAT explains over half of the effect attributable to systemic IR and adipose tissue dysfunction on CAC progression. These findings suggest that adiponectin and HOMA-IR could explain the increased cardiovascular risk associated with VAT. These results align with the hypothesis proposed by Reyes-Barrera J. et al., and Fan W. et al. suggesting that excessive production of free fatty acids and pro-inflammatory cytokines by ectopic fat depots may promote local damage, which leads to adipose tissue dysfunction [15, 16]. Additionally, in a previous report, we described that more than the excess in free fatty acids is the decreased concentration of circulating adiponectin, which explains the systemic IR, particularly among high VAT subjects [30]. Overall, our findings and previous evidence suggest that VAT should be interpreted as a marker of metabolic dysfunction driven by adipose tissue dysfunction and increased IR.

### METS-VF a VAT estimator that may be used daily in clinical practice

The METS-VF algorithm considers easily accessible biochemical and anthropometric evaluations to estimate VAT. METS-VF has been validated in the Mexican population and has been shown to be a good predictor of diabetes, hypertension, and metabolic syndrome [19, 35, 50]. The present study extends the utility of METS-VF by demonstrating that it is an adequate estimator of VAT tomographic measurement and showing that METS-VF behaved similarly to that of direct measurement of VAT in capturing the risk of CAC progression. Overall, our findings suggests that METS-VF could be used to identify subjects with a higher related adiposity risk of cardiovascular morbidity and mortality.

## Strengths and limitations

The present study’s strengths include a well-characterized prospective cohort of subjects without personal or familiar CAD history. These include the direct measurement of regional fat distribution by CT, the quantification of adiponectin, fasting insulin, and free fatty acid concentration. These variables allowed us to estimate ADIPO-IR and overall IR, all potential confounders of the VAT effect on CAC progression. Also, this study has some limitations. Since only include subjects with repeated coronary CT scans, there may be some selection bias. Furthermore, it is important to acknowledge that the estimated hazard ratios of VAT and METS-VF were relatively small, with values close to the unit, which may limit the clinical significance of the observed associations. It is essential to acknowledge that estimated hazard ratios for VAT and METS-VF were relatively small, which may limit the clinical relevance of the observed associations. Furthermore, other confounders, such as genetic background, lifestyle, and non-considered biochemical factors could influence the association between VAT and CAC progression. While our study provides valuable insights into the relationships between VAT, adipose tissue dysfunction markers, and CAC progression, further studies with larger sample sizes are warranted to confirm our findings and determine the clinical relevance of these markers in predicting CAC progression. The observational nature of this study may impact the accuracy of the medical history, dietary habits, and physical activity variables.

## Conclusion

In our Mexican cohort, we found that VAT is an independent predictor of CAC progression, being more evident among subjects classified as low risk by ASCVD score. Moreover, our study suggest that VAT is a mediator of the risk conferred by SAT dysfunction and IR. Finally, the METS-VF is an appropriate clinical surrogate of CT-VAT measurements, easily accessible in daily clinical practice, which could allow timely and effective evaluation of the cardiovascular risk conferred by VAT and associated dysfunctional adiposity.

## Supplementary Information

**Additional file 1: Table S1.** Impact of adiposity estimators on CAC progression. **Table S2.** Impact of the interaction of ASCVD, adiponectin, HOMA-IR, ADIPO-IR with VAT on CAC progression. **Table S3.** Impact of the interaction of ASCVD, adiponectin, HOMA-IR, ADIPO-IR with METS-VF on CAC progression. **Table S4.** Adjusted Cox proportional hazard regression models to assess the effect of HOMA-IR, VAT, ADIPO-IR and adiponectin related to CAC. **Table S5.** Adjusted Cox proportional hazard regression models to assess the effect of HOMA-IR, VAT, ADIPO-IR and adiponectin related to CAC adjusted for statin usage and ASCVD score.

**Additional file 2: (A)** Correlogram matrix of METS-VF with anthropometric and tomographic VAT measurements (CT-VAT). **(B)** AUROC of METS-VF, waist circumference, and BMI to detect increased VAT (CT-VAT ≥ p75).

## Data Availability

The data that support the findings of this study are available from the project administrator but restrictions apply to the availability of these data, which were used under license for the current study, and so are not publicly available. Data are however available from the authors upon reasonable request and with permission of the project administrator.

## Abbreviations

Adipo-IR: Adipose tissue insulin resistance
ASCVD: Atherosclerotic cardiovascular disease score
AT: Adipose tissue
BMI: Body mass index
CRF: Cardiovascular risk factors
CAC: Coronary artery calcium
CAD: Coronary artery disease
CI: Confidence interval
CT: Computed tomography
FFA: Free fatty acids
GEA: Genetics of Arteriosclerotic Disease study
HDL-C: High-density lipoprotein cholesterol
HOMA-IR: Homeostasis model for IR
HR: Hazard ratio
HU: Hounsfield units
IR: Insulin resistance
LDL-C: Low-density lipoprotein cholesterol
SAT: Subcutaneous adipose tissue
TC: Total cholesterol
TG: Triglycerides
VAT: Visceral adipose tissue
