# Supplementary material for: Visceral adipose tissue is an independent predictor and mediator of the progression of coronary calcification: a prospective sub-analysis of the GEA study
Source: Cardiovasc Diabetol. 2023 Apr 3;22:81. doi: 10.1186/s12933-023-01807-6 (PMC10071707; doi:10.1186/s12933-023-01807-6)
Supplement: Supplementary file 1 — Additional file 1: Table S1. Impact of adiposity estimators on CAC progression. Table S2. Impact of the interaction of ASCVD, adiponectin, HOMA-IR, ADIPO-IR with VAT on CAC progression. Table S3. Impact of the interaction of ASCVD, adiponectin, HOMA-IR, ADIPO-IR with METS-VF on CAC progression. Table S4. Adjusted Cox proportional hazard regression models to assess the effect of HOMA-IR, VAT, ADIPO-IR and adiponectin related to CAC. Table S5. Adjusted Cox proportional hazard regression models to assess the effect of HOMA-IR, VAT, ADIPO-IR and adiponectin related to CAC adjusted for statin usage and ASCVD score. [file 12933_2023_1807_MOESM1_ESM.pdf]

Table S1. Impact of adiposity estimators on CAC progression

	Unadjusted model		Model 1		Model 2		Model 3	
	**HR (95% CI)**	***p***	**HR (95% CI)**	***p***	**HR (95% CI)**	***p***	**HR (95% CI)**	***p***
BMI (Kg/m^2)	1.03(0.99-1.07)	0.07	1.03(0.99-1.07)	0.11	1.01(0.96-1.06)	NS	1.005(0.95-1.06)	NS
Waist circumference (cm)	1.03(1.02-1.04)	<0.0001	1.02(1.007-1.04)	<0.01	1.02(0.99-1.04)	0.08	1.014(0.99-1.03)	0.16
VAT (cm^2)	1.007(1.004-1.009)	<0.0001	1.005(1.003-1.008)	<0.0001	1.004(1.001-1.007)	<0.01	1.004(1.001-1.007)	<0.01
METS-VF*	1.001(1.001-1.002)	<0.0001	1.001(1.0-1.001)	0.001	1.001(1.0-1.001)	<0.05	1.003(1.0-1.001)	<0.05

BMI: body mass index, VAT: visceral adipose tissue *METS-VF: metabolic score for visceral fat estimation exponentially transformed to express VAT in grams. Model 1: ASCVD and statin use adjusted, Model 2: Model 1, plus HOMA-RI and adiponectin adjusted. Model 3: Model 2, plus C-reactive protein, hypertension status and dyslipidemia status. Cox proportional-hazard regression analysis.

Table S2. Impact of the interaction of ASCVD, adiponectin, HOMA-IR, ADIPO-IR with VAT on CAC progression

	**HR (CI 95%)**	***p***	**Model 1[#]**	***p***
Individual effect				
VAT	1.007(1.004-1.009)	<0.0001	1.007(1.004-1.009)	<0.0001
ASCVD	1.08(1.06-1.1)	<0.0001	1.08(1.06-1.1)	<0.0001
Adiponectin	0.96(0.94-0.99)	<0.05	0.96(0.94-0.99)	<0.05
HOMA	1.1(1.04-1.16)	NS	1.1(1.04-1.16)	<0.01
ADIPO-IR	1.006(0.99-1.02)	NS	1.006(0.99-1.02)	NS
**ASCVD interaction**				
VAT alone	1.007(1.004-1.009)	<0.0001	1.009(1.005-1.01)	<0.0001
ASCVD alone	1.17(1.09-1.26)	<0.0001	1.18(1.1-1.27)	<0.0001
VAT *ASCVD interaction	1.0(0.99-1.0)	<0.01	1.0(0.99-1.0)	<0.01
**Stratified by ASCVD risk[§]**				
Low	1.008(1.004-1.012)	<0.01		
Medium	1.003(0.99-1.008)	NS		
High	1.003(0.99-1.006)	NS		
Adiponectin interaction				
VAT alone	1.007(1.002-1.01)	<0.01		
Adiponectin alone	0.99(0.93-1.07)	NS		
VAT *Adiponectin interaction	1.0(0.99-1.0)	NS		
HOMA-IR interaction				
VAT	1.005(1.0-1.01)	<0.05		
HOMA-IR	0.99(0.81-1.22)	NS		
VAT *HOMA-IR interaction	1.0(0.99-1.0)	NS		
ADIPO-IR interaction				
VAT	1.005(1.002-1.009)	0.001		
ADIPO-IR	0.99(0.96-1.03)	NS		
VAT *ADIPO-IR interaction	1.0(1.0-1.0)	NS		

VAT: visceral adipose tissue, ASCVD: atherosclerotic cardiovascular disease risk score. HOMA-IR: homeostatic model for insulin resistance, ADIPO-IR: adipose tissue insulin resistance.

[#] Statin use adjusted, Cox regression analysis, [§]Model 1 stratified by ASCVD risk

Table S3. Impact of the interaction of ASCVD, adiponectin, HOMA-IR, ADIPO-IR with METS-VF on CAC progression

	HR (IC 95%)	*p*	Model 1[#]	*p*
Individual effect				
METS-VF	1.001(1.001-1.002)	<0.0001	1.001(1.001-1.002)	<0.0001
ASCVD	1.08(1.06-1.1)	<0.0001	1.08(1.06-1.1)	<0.0001
Adiponectin	0.96(0.94-0.99)	<0.05	0.96(0.94-0.99)	<0.05
HOMA-IR	1.1(1.04-1.16)	NS	1.1(1.04-1.16)	<0.01
ADIPO-IR	1.006(0.99-1.02)	NS	1.006(0.99-1.02)	NS
ASCVD interaction				
METS-VF	1.001(1.001-1.002)	<0.0001	1.001(1.001-1.002)	<0.0001
ASCVD	1.18(1.09-1.27)	<0.0001	1.18(1.09-1.28)	<0.0001
METS-VF* ASCVD interaction	1.0(1.0-1.0)	0.011	1.0(1.0-1.0)	0.011
**Stratified by ASCVD risk[§]**				
Low	1.001(1.00-1.022)	0.01		
Medium	1.001(1.00-1.002)	0.01		
High	1.00 (1.00-1.001)	0.01		
Adiponectin interaction				
METS-VF	1.001(1.0-1.001)	0.015		
Adiponectin	0.96(0.89-1.04)	NS		
METS-VF*Adiponectin interaction	1.0(1.0-1.0)	NS		
HOMA-IR interaction				
METS-VF	1.001(1.001-1.002)	0.012		
HOMA-IR	0.99(0.78-1.26)	NS		
METS-VF*HOMA-IR interaction	1.0(1.0-1.0)	NS		
ADIPO-IR interaction				
METS-VF	1.001(1.001-1.002)	<0.0001		
ADIPO-IR	0.99(0.96-1.03)	NS		
METS-VF*ADIPO-IR interaction	1.0(1.0-1.0)	NS		

METS-VF: metabolic score for visceral fat estimation exponentially transformed to express VAT in grams, ASCVD: atherosclerotic cardiovascular disease risk score. HOMA-IR: homeostatic model for insulin resistance, ADIPO-IR: adipose tissue insulin resistance.

[#] Statin use adjusted, Cox regression analysis, [§]Model 1 stratified by ASCVD risk

Table S4. Adjusted Cox proportional hazard regression models to assess the effect of HOMA-IR, VAT, ADIPO-IR and adiponectin related to CAC

**Steep**	**Variables in the model**		**Coefficient**	**HR**	**SE**	**p**	
**1 Univariate relation**	HOMA-IR	HOMA-IR	0.095	1.099	0.029	<0.001*	-
	ADIPO-IR	ADIPO-IR	0.007	1.007	0.005	0.193	-
	Adiponectin	Adiponectin	-0.040	0.961	0.014	0.006*	-
**2 Double combination**	HOMA-IR + Adiponectin	HOMA-IR	0.077	1.080	0.030	0.012	1.056
		Adiponectin	-0.033	1.006	0.001	<0.001*	1.056
	HOMA-IR + ADIPO-IR	HOMA-IR	0.091	1.095	0.030	<0.001*	1.056
		ADIPO-IR	0.004	1.004	0.007	0.592	1.056
	Adiponectin + ADIPO-IR	Adiponectin	-0.039	0.961	0.014	<0.001*	1.001
		ADIPO-IR	0.006	1.006	0.005	0.254	1.001
**3 Double combination VAT adjusted**	HOMA-IR + Adiponectin + VAT	HOMA-IR	0.042	1.043	0.033	0.210	1.094
		Adiponectin	-0.020	0.980	0.015	0.169	1.077
		VAT	0.006	1.006	0.001	<0.001*	1.111
	HOMA-IR + ADIPO-IR + VAT	HOMA-IR	0.045	1.046	0.034	0.189	1.142
		ADIPO-IR	0.004	1.004	0.008	0.596	1.063
		VAT	0.006	1.006	0.001	<0.001*	1.077
	Adiponectin + ADIPO-IR + VAT	Adiponectin	-0.023	0.978	0.015	0.128	1.056
		ADIPO-IR	0.006	1.006	0.007	0.391	1.002
		VAT	0.006	1.006	0.001	<0.001*	1.055

Table S5. Adjusted Cox proportional hazard regression models to assess the effect of HOMA-IR, VAT, ADIPO-IR and adiponectin related to CAC adjusted for statin usage and ASCVD score

Steep	Variables in the model		Coefficient	HR	SE	p	VIF
1<br>Univariate relation	HOMA-IR	HOMA-IR	0.055	1.057	0.034	0.099†	-
	ADIPO-IR	ADIPO-IR	0.004	1.004	0.007	0.590	-
	Adiponectin	Adiponectin	-0.029	0.971	0.015	0.043*	-
2<br>Double combination	HOMA-IR + Adiponectin	HOMA-IR	0.055	1.057	0.034	0.099†	1.079
		Adiponectin	-0.029	0.971	0.015	0.043*	1.070
	HOMA-IR + ADIPO-IR	HOMA-IR	0.051	1.052	0.035	0.142	1.143
		ADIPO-IR	0.004	1.004	0.007	0.590	1.064
	Adiponectin + ADIPO-IR	Adiponectin	-0.030	0.971	0.015	0.042*	1.070
		ADIPO-IR	0.004	1.004	0.007	0.590	1.064
3<br>Double combination VAT adjusted	HOMA-IR + Adiponectin + VAT	HOMA-IR	0.037	1.038	0.036	0.297	1.130
		Adiponectin	-0.023	0.977	0.015	0.124	1.104
		VAT	0.004	1.004	0.001	0.016*	1.275
	HOMA-IR + ADIPO-IR + VAT	HOMA-IR	0.033	1.034	0.037	0.370	1.202
		ADIPO-IR	0.004	1.004	0.008	0.644	1.078
		VAT	0.004	1.004	0.001	0.017*	1.275
	Adiponectin + ADIPO-IR + VAT	Adiponectin	-0.023	0.977	0.015	0.121	1.105
		ADIPO-IR	0.004	1.004	0.008	0.644	1.078
		VAT	0.004	1.004	0.001	0.017*	1.275
