# Supplementary figures and images for: Visceral adipose tissue is an independent predictor and mediator of the progression of coronary calcification: a prospective sub-analysis of the GEA study
Source: Cardiovasc Diabetol. 2023 Apr 3;22:81. doi: 10.1186/s12933-023-01807-6 (PMC10071707; doi:10.1186/s12933-023-01807-6)

A)

Spearman Correlation Analysis of Adiposity Measurements

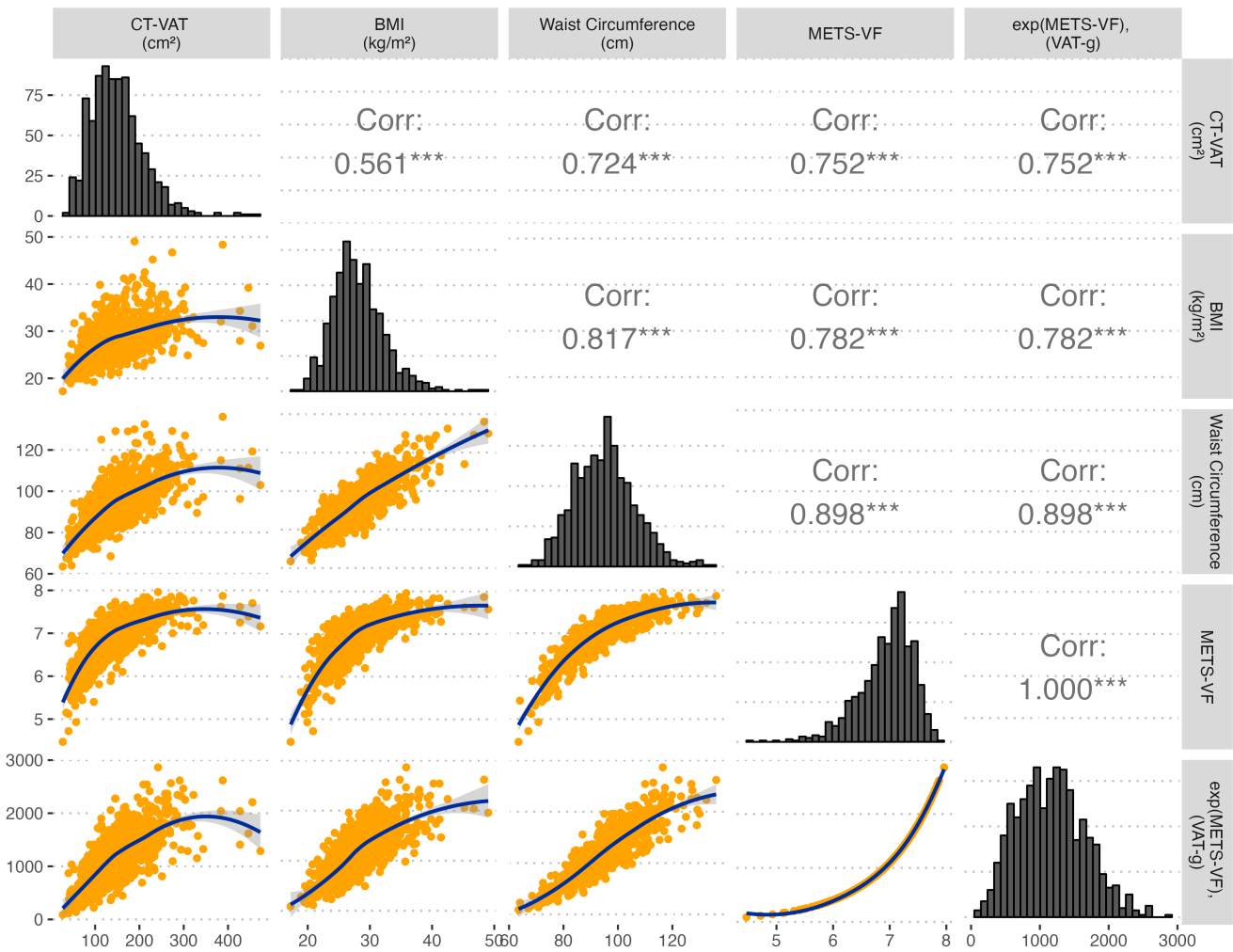

B)

AUROC to Detect VAT  $\geq$  75

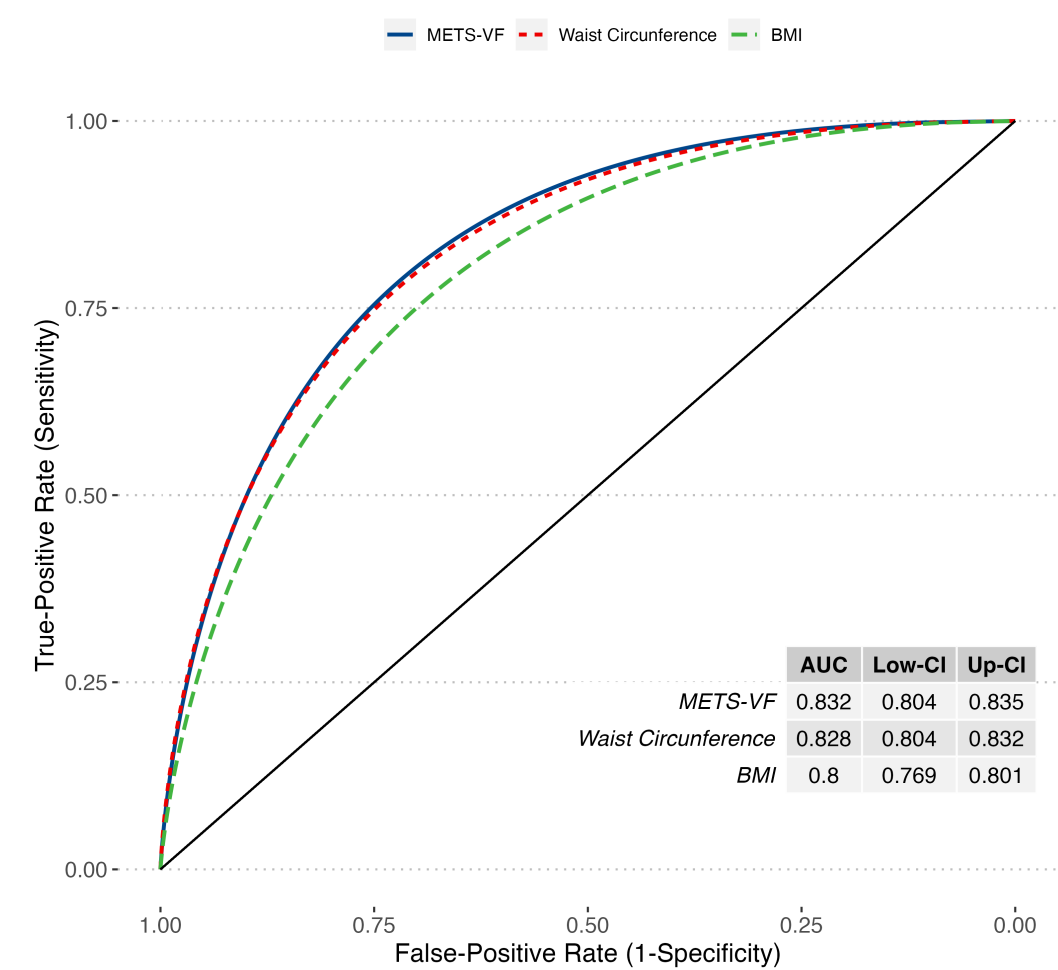

Supplement: Supplementary file 2 — Additional file 2: (A) Correlogram matrix of METS-VF with anthropometric and tomographic VAT measurements (CT-VAT). (B) AUROC of METS-VF, waist circumference, and BMI to detect increased VAT (CT-VAT ≥ p75). [file 12933_2023_1807_MOESM2_ESM.pdf]
